# Aberrations in *FGFR1*, *FGFR2*, and RIP5 Expression in Human Congenital Anomalies of the Kidney and Urinary Tract (CAKUT)

**DOI:** 10.3390/ijms232415537

**Published:** 2022-12-08

**Authors:** Nela Kelam, Anita Racetin, Mirjana Polović, Benjamin Benzon, Marin Ogorevc, Katarina Vukojević, Merica Glavina Durdov, Ana Dunatov Huljev, Ivana Kuzmić Prusac, Davor Čarić, Fila Raguž, Sandra Kostić

**Affiliations:** 1 Department of Anatomy, Histology and Embryology, University of Split School of Medicine, Šoltanska 2, 21000 Split, Croatia; 2 Department of Anatomy, University of Mostar School of Medicine, 88000 Mostar, Bosnia and Herzegovina; 3 Department of Pathology, University Hospital Center Split, 21000 Split, Croatia; 4 Department of Orthopaedics and Traumatology, University Hospital in Split, Spinciceva 1, 21000 Split, Croatia; 5 Department of Nephrology, University Hospital Center Mostar, 88000 Mostar, Bosnia and Herzegovina

**Keywords:** RIP5, *FGFR1*, *FGFR2*, kidney development, congenital anomalies of the kidney and urinary tract, CAKUT

## Abstract

This study aimed to explore the spatio-temporal expression patterns of congenital anomalies of kidney and urinary tract (CAKUT) candidate genes, Fibroblast Growth Factor Receptor 1 (FGFR1), Fibroblast Growth Factor Receptor 2 (FGFR2) and Receptor-Interacting Protein Kinase 5 (RIP5), in human fetal kidney development (CTRL) and kidneys affected with CAKUT. Human fetal kidneys from the 22nd to 41st developmental week (duplex, hypoplastic, dysplastic, and controls) were stained with antibodies and analyzed by epifluorescence microscopy and RT−qPCR. The effect of CAKUT candidate genes on kidney nephrogenesis and function is confirmed by statistically significant variations in the spatio-temporal expression patterns of the investigated markers. The nuclear localization of FGFR1, elevated expression score of *FGFR1* mRNA, the increased area percentage of FGFR1-positive cells in the kidney cortex, and the overall decrease in the expression after the peak at the 27th developmental week in dysplastic kidneys (DYS), suggest an altered expression pattern and protein function in response to CAKUT pathophysiology. The RT−qPCR analysis revealed a significantly higher *FGFR2* mRNA expression score in the CAKUT kidneys compared to the CTRL. This increase could be due to the repair mechanism involving the downstream mediator, Extracellular Signal-Regulated Kinase 1/2 (ERK1/2). The expression of RIP5 during normal human kidney development was reduced temporarily, due to urine production and increased later since it undertakes additional functions in the maturation of the postnatal kidney and homeostasis, while the expression dynamics in CAKUT-affected kidneys exhibited a decrease in the percentage of RIP5-positive cells during the investigated developmental period. Our findings highlight the importance of FGFR1, FGFR2, and RIP5 as markers in normal and pathological kidney development.

## 1. Introduction

Obstructions in the kidney and urinary tract’s embryonic development result in a range of structural abnormalities labeled as congenital anomalies of the kidney and urinary tract (CAKUT). CAKUT contributes to 23% of birth abnormalities [1,2] and accounts for approximately 50% of pediatric cases and 7% of adult cases of end-stage renal disease worldwide [3,4]. The phenotypic spectrum of CAKUT is very diverse. It includes variable degrees of parenchymal kidney defects (renal agenesis, hypoplasia, dysplasia, or multicystic dysplastic kidney), upper urinary tract obstructions (ureteropelvic junction obstruction (UPJO), obstructive and refluxing megaureter, or vesicoureteral reflux (VUR)), and lower urinary tract defects (posterior urethral valves). The severity of CAKUT also varies greatly, ranging from benign conditions like an ectopic kidney to fatal diseases such as bilateral multicystic dysplastic kidney or bilateral kidney agenesis [1,5,6]. 

To date, monogenic abnormalities associated with CAKUT involve mutations in 23 genes and have been detected in just a small percentage (12%) of investigated cases, with inadequate evidence of their causal involvement [7]. Some of these mutations are detected in Fibroblast Growth Factor Receptors (FGFRs) and substrates of their signaling transduction pathways, one of them being Receptor-Interacting Protein Kinase 5 (RIP5, DSTYK) [8,9,10].

In the fetal kidney, RIP5 and FGFRs are co-expressed in the metanephric mesenchyme and ureteric bud, thus making RIP5 a critical regulator of human urinary system development downstream of Fibroblast Growth Factor (FGF) signaling [9,11,12,13]. In human embryonic kidney cell culture, RIP5 knockdown inhibits FGF-stimulated phosphorylation of Extracellular Signal-Regulated Kinase (ERK), the most important signal downstream of receptor tyrosine kinases [9]. Interestingly, in a recent study of seven affected family members with CAKUT, disease-causing mutations were identified in the *RIP5* gene. Additional *RIP5* mutations were detected in seven out of 311 (2.3%) unrelated patients with congenital kidney or urinary tract abnormalities, such as renal hypoplasia and UPJO, suggesting *RIP5* is a novel CAKUT gene [9,14].

FGFR signaling is critical for patterning practically all renal lineages at different stages of development [10]. The impairment of the FGF/FGFR signaling axis is detected in numerous human syndromes and diseases, including chronic kidney disease (CKD) [9,15]. Activating mutations of *FGFR2* (or rarely *FGFR1*) can cause several syndromes: Apert, Pfeiffer, Antley–Bixler, or Beare–Stevens syndrome, which may be coupled with a phenotype belonging to the CAKUT spectrum: hydroureter, unilateral renal aplasia, or vesicoureteral reflux [16,17,18,19].

Different genetic *Fgfr* and *Fgf* animal models have morphological defects resembling a variety of CAKUTs seen in humans [15,20,21,22,23,24]. In mice, deletion of *Fgfr1* or *Fgfr2* causes early death of the embryo prior to the onset of renal development [10,25,26,27,28]. Global deletion of *Fgfr1* disrupts nephron formation, whereas loss of *Fgfr2* in the metanephric mesenchyme, essential for ureteric morphogenesis, results in several kidney and urinary tract defects [23]. Postnatally, renal hypoplasia in *Fgfr2* mutants resulted in hypertension, CKD, and left ventricular hypertrophy [29]. Transgenic mice with a dominant-negative *Fgfr* fragment, develop renal aplasia or severe dysplasia [30]. 

Aberrations in molecular, cellular, and morphogenetic mechanisms that direct renal system development are major causes of CAKUT. A thorough understanding of how morphogenetic process disruptions are related to CAKUT would improve the diagnosis, treatment, and prevention of congenital renal system defects and their repercussions [31]. 

We analyzed the spatio-temporal expression pattern of *FGFR1*, *FGFR2*, and *RIP5* in normal human kidney development and the development of CAKUT-affected kidneys from the 22nd to 41st developmental week (dw), to determine how they are related to the occurrence of CAKUT. These genes have been selected as CAKUT candidate genes. Current knowledge on the topic is mainly based on gene-targeting studies in knockout mice. The results of our study on human material might have meaningful implications for a better understanding of normal human kidney development, the development of CAKUT, and the possible therapeutic potential of investigated gene products and their signaling pathways.

## 2. Results

Morphological differences between normal human kidneys and kidneys with CAKUT were observed in H&E slides. In immunofluorescence, expression of FGFR1, FGFR2, and RIP5 was evaluated in tubules, collecting ducts, and developing glomeruli, with different intensity and expression patterns. Quantitative cell evaluation of immunoreactivity of FGFR1, FGFR2, and RIP5 in normal and CAKUT kidneys was conducted by determining the section percentage area of the fetal kidney cortex, both in the nephrogenic zone and juxtamedullary region. Results were expressed as area percentages of the positive signal. Furthermore, linear and nonlinear regression modeling was used to study expression dynamics and progression of FGFR1, FGFR2, and RIP5 throughout the observed developmental periods. RT−qPCR analysis of FFPE kidney tissues, including both the cortex and medullary region, was performed on the same specimens to determine the expression score of observed mRNAs between CAKUT-affected kidneys and CTRL as well as between different CAKUT phenotypes (DU, HYP, DYS) and CTRL.

### 2.1. H&E Staining of Normal Human Fetal Kidney and Kidneys with Congenital Anomalies of the Kidney and Urinary Tract (CAKUT)

**Figure 1:**
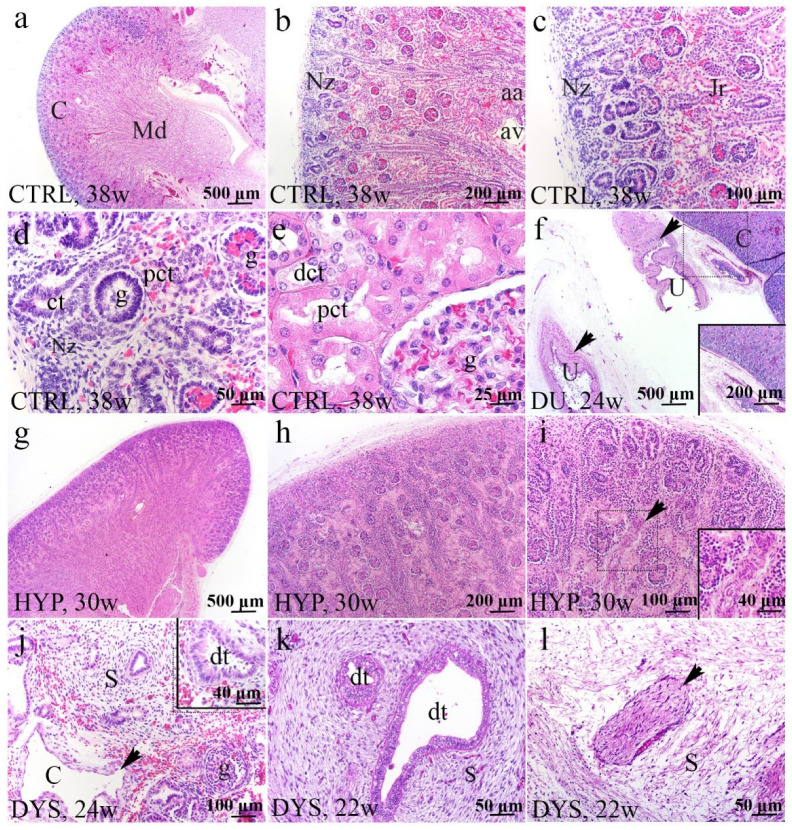
Histology of normal fetal kidney at 38th dw (**a**–**e**) and kidneys affected with congenital anomalies: duplex kidney (DU) at 24th dw, hypoplastic kidneys (HYP) at 30th dw, and dysplastic kidney (DYS) at 22nd and 24th dw (H&E) (**f**–**l**). Cortex (C) and medullary pyramid (Md) in the normal kidney at 38th dw (**a**). The thin nephrogenic zone (Nz) is a basophilic band of developing nephrons in the outermost portion of the cortex; arcuate artery (aa), arcuate vein (av), juxtamedullary region (Jr) (**b**,**c**). The stages of individual nephron development; glomeruli (g), convoluted tubule (ct), proximal convoluted tubule (pct) (**d**). Tubular growth and maturation (**e**). Duplex kidney segments with two ureters (U) (arrows) and normal renal parenchyma on 24th dw (**f**). The hypoplastic renal cortex had very thin Nz on 30th dw (**g**). The hypoplastic kidney has a thin cortex with a reduced number of nephrons (**h**). The Nz in the hypoplastic kidney was thinner and characterized by a thick-walled artery. The arrow shows an artery of the unusual, tortuous form (**i**). Renal dysplasia (DYS) was characterized by many thin-walled cysts (arrow), cystically dilated proximal tubules, undifferentiated stroma (S), and a few glomeruli (g) (**j**). Dysplastic tubules (dt) were lined by tall columnar epithelium surrounded by stromal spindle cells arranged circumferentially as a collar (**k**). Large peripheral nerves (arrow) were embedded in the kidney mesenchyme (**l**). The most characteristic elements noticed for each phenotype are shown in inserts corresponding to the dashed boxes. Images were taken at a magnification of ×2 (**a**,**f**,**g**), ×4 (**b**,**h**), ×10 (**c**,**i**,**j**), ×20 (**d**,**k**,**l**), and ×40 (**e**).

The normal fetal kidney had a partly different histological appearance than the adult kidney, because it develops until the end of gestation when nephrogenesis terminates. The normal human kidney was organized in lobes consisting of a medullary pyramid (Md) and surrounding cortex (C), both primarily formed in the nephrogenic zone (Nz) (Figure 1a,b). Under the capsule, Nz was observable as a basophilic band of developing nephrons in the outermost portion of the cortex, revealing the stages of individual nephron development (Figure 1c). The most recently formed glomeruli enlarged as they integrated into the expanding cortex underneath the nephrogenic zone (Figure 1c). The young glomeruli were more densely packed in the outer cortex, while older glomeruli were more widely separated and situated near the medulla due to tubular elongation and widening (Figure 1b). Glomeruli are differentiated on one end of the developing nephron, and tubular differentiation was recognized relatively early. Proximal convoluted tubules (pct) were easily recognizable beneath the nephrogenic zone due to cytoplasmic eosinophilia (Figure 1d). Fetal glomeruli were smaller than adult glomeruli, and the podocytes had a characteristic cuboidal appearance (Figure 1e). 

Duplex kidneys, a common abnormality of renal tract development, presented as a renal unit comprised of two pelvicalyceal systems but demonstrated a normal microscopic appearance (Figure 1f). 

Kidneys diagnosed with renal hypoplasia exhibited normal cortico-medullar organization but were smaller in size, with a lower number of nephrons and calyces than the normal kidney (Figure 1g). The renal cortex was thin, with an arrest in the glomerular migration toward the inner zone of the cortex (Figure 1h). There were large, dilated veins and thick-walled tortuous arteries. Cysts, atrophic tubules, and other dysplastic features were absent (Figure 1i).

Dysplastic kidneys result from abnormal inductive interactions between epithelial cells in the ampulla/ureteric bud and surrounding mesenchyme. This leads to aberrant development and branching of collecting ducts, loss of functioning nephrons, and formation of aberrant structures, including dysplastic tubules, cysts derived from primitive ducts, stromal expansion, and proliferation of peripheral nerves (Figure 1j–l). Many thin-walled cysts were scattered throughout the renal parenchyma (Figure 1j). Dysplastic tubules with irregular outlines predominated in the outer cortex and were surrounded by collarettes of smooth muscle-like undifferentiated mesenchyme (Figure 1k). Large trunks of nerve tissue embedded in the kidney mesenchyme predominantly consisted of unmyelinated nerve fibers (Figure 1l).

### 2.2. FGFR1 Expression

**Figure 2:**
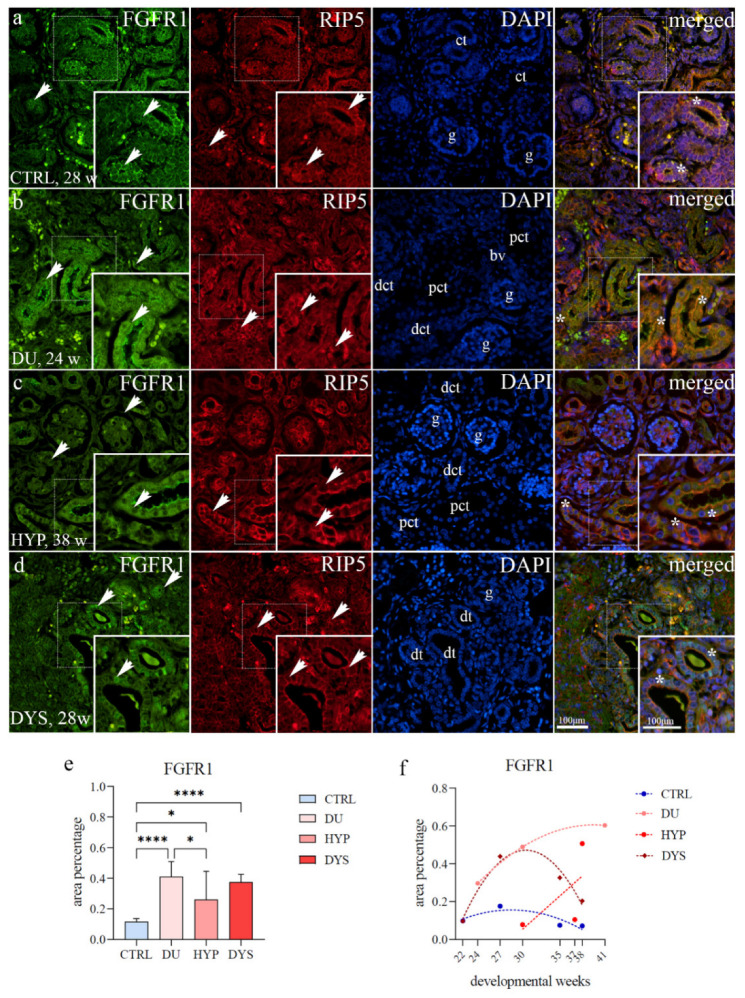
Double immunofluorescence staining of human fetal kidneys with the antibodies for FGFR1 and RIP5 (**a**–**d**). Arrows show the expression pattern of FGFR1 and RIP5 in glomeruli (g), convoluted tubules (ct), proximal convoluted tubules (pct), distal convoluted tubules (dct), blood vessels (bv), dysplastic tubules (dt), indicated on the DAPI image. Immunoexpression of FGFR1, RIP5, DAPI staining and merged FGFR1, RIP5, and DAPI in control (CTRL) at 28th dw (**a**), duplex kidney (DU) at 24th dw (**b**), hypoplastic kidney (HYP) at 38th dw (**c**) and dysplastic kidney (DYS) at 28th dw (**d**). The most prominent protein expression area is shown in inserts corresponding to the dashed boxes. An asterisk denotes the zone where co-expression was detected. Images were taken at a magnification of ×40. The scale bar is 100 μm, which refers to all images. The FGFR1 area percentages in the cortex of CTRL, DU, HYP, and DYS fetal kidney tissues (**e**). Data are presented as the mean ± SD (vertical line) and analyzed by an ordinary one-way ANOVA test followed by Tukey’s multiple comparison test. Significant differences were indicated by * *p* < 0.05, **** *p* < 0.00001. At each time point, ten representative pictures were assessed. The expression dynamics of FGFR1 (**f**) showed by linear and nonlinear regression modeling of area percentages through developmental periods in the cortex of CTRL, DU, HYP, and DYS fetal kidney tissues at the 22nd, 24th, 27th, 30th, 35th, 37th, 38th, and 41st dw. Data are presented as the mean ± SEM.

The immunohistochemical staining pattern of FGFR1 within the positive tubular segments was not uniform, but ranged from negative to membranous and diffuse cytoplasmic staining. Mild punctate expression was found in the apical membrane of collecting ducts, including ampullae and developing nephrons (metanephric cup, renal vesicle stages) and convoluted tubules in the nephrogenic zone of healthy controls. In the convoluted tubules and parietal glomerular epithelial cells of immature glomeruli of the juxtamedullary region, a diffuse FGFR1 expression pattern was noticed (Figure 2a). Endothelial cells of arteries were FGFR1 positive in the healthy controls and CAKUT-affected kidneys (Figure 2b).

The CAKUT-affected kidneys have shown strong diffuse staining of apical membranes of proximal convoluted tubules (Figure 2b,d). In the DYS, localization of FGFR1 in the nuclei of epithelial cells of convoluted tubules was found. Co-expression of FGFR1 and RIP5 was present in different substructures and found in the epithelial cells of convoluted tubules in all observed phenotypes (Figure 2a–d).

The area percentage of FGFR1-positive cells in controls, showed a significant decrease compared to DU (*p* < 0.0001), HYP (*p* = 0.0228), and DYS (*p* < 0.0001) (F (3, 36) = 15,27; Figure 2e). FGFR1 expression is higher in DU than in HYP (*p* = 0.0186, Figure 2e).

In HYP kidneys, the expression of FGFR1 increased during fetal development. No significant difference was found when formally tested for a linear trend among developmental periods (R^2^ = 40.83%, β = 0.035 ± 0.007, Figure 2f). The overall expression of FGFR1 in DU showed exponential growth with the developmental age, with peak expression at 41 weeks (R^2^ = 100%). The FGFR1 expression in CTRL and DYS followed a quadratic trend (R^2^ = 71.75% and 95.49%) with peak expression at 27 dw.

**Figure 3:**
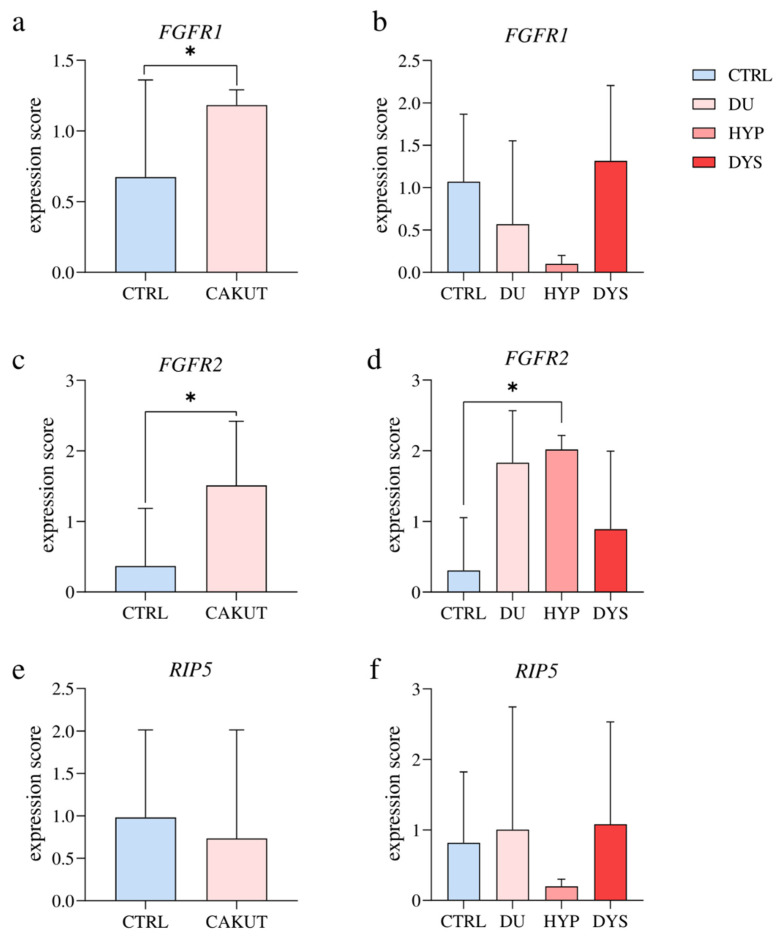
The RT−qPCR analysis of healthy human fetal and CAKUT-affected kidneys using primers for *FGFR1* (**a**,**b**), *FGFR2* (**c**,**d**), and *RIP5* (**e**,**f**). The *FGFR1* mRNA expression score comparison between the group of CAKUT-affected kidneys (CAKUT) and the healthy control group (CTRL). Data are analyzed by unpaired *t*-test (**a**). The *FGFR1* mRNA expression score comparison between different CAKUT phenotypes: duplex kidneys (DU), hypoplastic kidneys (HYP), dysplastic kidneys (DYS), and CTRL. Ordinary one-way ANOVA followed by Tukey’s multiple comparison test (**b**). The *FGFR2* mRNA expression score comparison between CAKUT and CTRL. Unpaired *t*-test with Welch’s correction (**c**). The *FGFR2* mRNA expression score comparison between DU, HYP, DYS, and CTRL. Ordinary one-way ANOVA, followed by Tukey’s multiple comparison test (**d**). The *RIP5* mRNA expression score comparison between CAKUT and CTRL. Unpaired *t*-test (**e**). The *RIP5* mRNA expression score comparison between DU, HYP, DYS, and CTRL. Ordinary one-way ANOVA followed by Tukey’s multiple comparison test (**f**). Data are presented as the mean ± SD (vertical line); significant differences were indicated by * *p* < 0.05.

The RT−qPCR analysis revealed a significantly higher *FGFR1* expression score in the CAKUT kidneys than in the controls (*p* = 0.0339, F (4, 9) = 40,13; Figure 3a). No significant difference was observed when we compared the *FGFR1* mRNA expression in different CAKUT phenotypes to controls (*p* = 0.2412, F (3, 11) = 1,619; Figure 3b).

### 2.3. FGFR2 Expression

**Figure 4:**
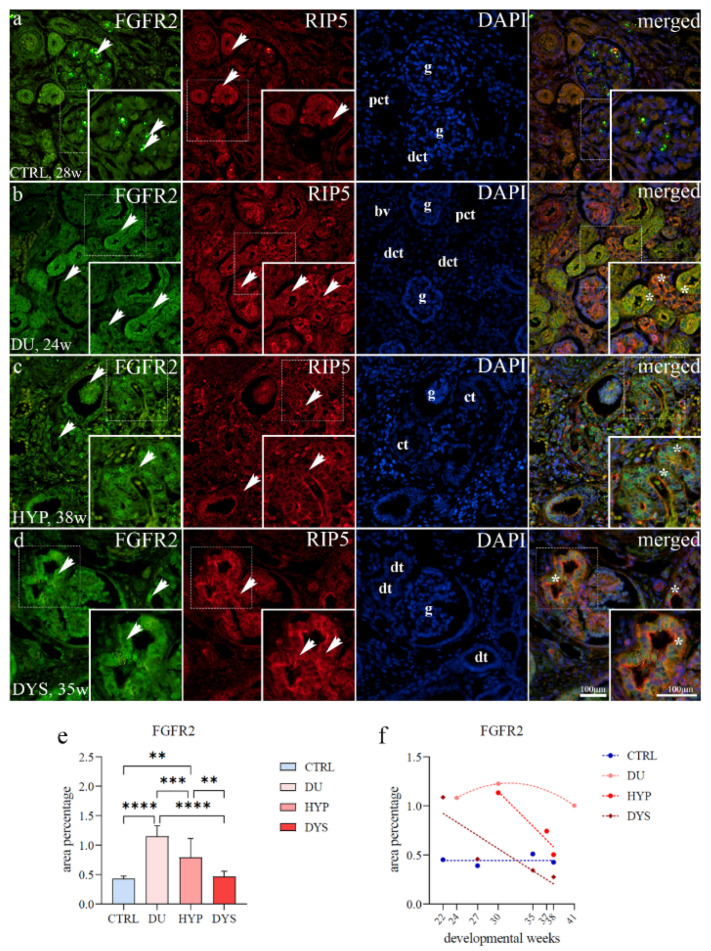
Double immunofluorescence staining of human fetal kidneys with the antibodies for FGFR2 and RIP5 (**a**–**d**). Arrows show the expression pattern of FGFR2 and RIP5 in glomeruli (g), convoluted tubules (ct), proximal convoluted tubules (pct), distal convoluted tubules (dct), blood vessels (bv), dysplastic tubules (dt) indicated on DAPI image. Immunoexpression of FGFR2, RIP5, DAPI staining and merged FGFR2, RIP5, and DAPI in control (CTRL) at 28th dw (**a**), duplex kidney (DU) at 24th dw (**b**), hypoplastic kidney (HYP) at 38th dw (**c**) and dysplastic kidney (DYS) at 35th dw (**d**). The most prominent protein expression area is shown in inserts corresponding to the dashed boxes. An asterisk denotes the zone where co-expression was detected. Images were taken at a magnification of ×40. The scale bar is 100 μm, which refers to all images. The FGFR2 (**e**) area percentages in the cortex of CTRL, DU, HYP, and DYS fetal kidney tissues. Data are presented as the mean ± SD (vertical line) and analyzed by an ordinary one-way ANOVA test followed by Tukey’s multiple comparison test. Significant differences were indicated by ** *p* < 0.01, *** *p* < 0.001, **** *p* < 0.0001. At each time point, ten representative pictures were assessed. The expression dynamics of FGFR2 (**f**) showed by linear and nonlinear regression modeling of area percentages through developmental periods in the cortex of CTRL, DU, HYP, and DYS fetal kidney tissues at the 22nd, 24th, 27th, 30th, 35th, 37th, 38th, and 41st dw. Data are presented as the mean ± SEM.

FGFR2 showed strong staining of the apical epithelial membrane of convoluted tubules, sometimes within the basolateral membrane and in developing nephrons within the nephrogenic zone. In the juxtamedullary region, an abundance of strong diffuse staining of the proximal convoluted tubules and punctate staining of visceral glomerular epithelial cells of immature glomeruli can be observed (Figure 4a).

The CAKUT-affected kidneys displayed strong, diffuse membranous and cytoplasmic staining patterns in convoluted tubules, endothelial cells of blood vessels, and parietal epithelial glomerular cells of Bowman’s capsule (Figure 4b–d). In the DU and HYP, we noticed localization of FGFR2 in the nuclei of epithelial cells of convoluted tubules (Figure 4b,c).

Merging the expression of FGFR2 and RIP5, their co-expression was found in glomeruli and proximal and distal convoluted tubules (Figure 4b–d).

The area percentage of FGFR2-positive cells significantly differed between CAKUT-affected kidneys and controls (F (3, 36) = 30,10; Figure 4f). The significantly highest area percentage was noticed in DU compared to DYS, CTRL (*p* < 0.0001) and HYP (*p* = 0.0010). HYP kidneys demonstrated significantly higher area percentage scores than DYS (*p* = 0.0034) and CTRL (0.0011).

The percentage of FGFR2-positive cells in HYP and DYS kidneys gradually reduced over time. When formally tested for a linear trend among developmental periods, the analysis did not show significance (R^2^ = 93.03%, β = −0.07 ± 0.04; R^2^ = 78.38%, β = −0.04 ± 0.02, Figure 4f). The overall expression of FGFR2 with the developmental age in DU demonstrated a quadratic trend, with peak expression at 30 weeks (R^2^ = 100%). However, CTRL demonstrated a positive linear trend (R^2^ = 96.03%, β = 0.002 ± 0.006).

The RT−qPCR analysis revealed a significantly higher *FGFR2* expression score in the CAKUT kidneys than in the control (*p* = 0.0364, F (9, 4) = 1,232; Figure 3c). When we compared the *FGFR2* mRNA expression in different CAKUT phenotypes to controls, we detected a significantly higher expression score in HYP than in CTRL (*p* = 0.0441, F (3, 12) = 4,240; Figure 3d).

### 2.4. RIP5 Expression

RIP5 demonstrated continuous expression, regardless of the developmental stages or phenotypes of the analyzed samples. RIP5-positive cells were recognized as red fluorescence in the apical and basolateral membranes. Cytoplasmatic staining was noted in diffuse and punctate forms (Figure 4a,b).

In the nephrogenic zone of the kidney cortex, RIP5 was moderately expressed in the endothelium of blood vessels, developing nephrons (renal vesicle stages, metanephric cup), and epithelium of collecting ducts, including ampullae. Also, compared to expression in renal vesicle stages, a more robust expression pattern was discovered in the juvenile glomeruli, notably in the parietal layer of the Bowman’s capsule (Figure 2c).

Strong RIP5 expression was seen in the apical cytoplasm of the proximal and distal tubules (Figure 2b,d) and the endothelium of blood vessels (Figure 2b,d and Figure 4b), in the juxtaglomerular area, but very weakly in the glomeruli, with notable staining in podocytes (Figure 2d and Figure 4b). RIP5 co-expressed with FGFR1 and FGFR2 in the control and CAKUT-affected kidney substructures.

**Figure 5:**
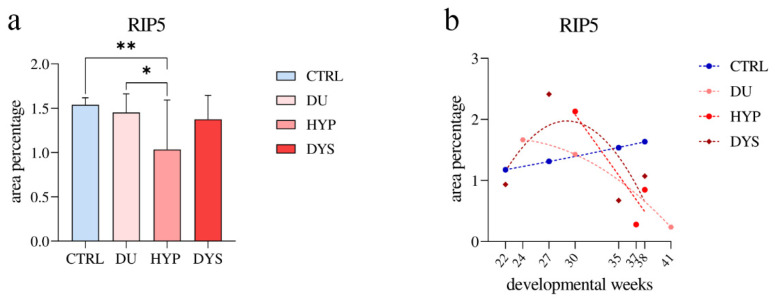
The RIP5 area percentages in the controls (CTRL), duplex kidneys (DU), hypoplastic kidneys (HYP), and dysplastic kidneys (DYS) in the fetal tissue cortex. Data are presented as the mean ± SD (vertical line) and analyzed by an ordinary one-way ANOVA test followed by Tukey’s multiple comparison test. Significant differences were indicated by * *p* < 0.05, ** *p* < 0.01 (**a**). The expression dynamics of FGFR2 are shown by linear and nonlinear regression modeling of area percentages through developmental periods in the cortex of CTRL, DU, HYP, and DYS fetal kidney tissues at the 22nd, 24th, 27th, 30th, 35th, 37th, 38th, and 41st dw. Data are presented as the mean ± SEM (**b**). At each time point, ten representative pictures were assessed.

The area percentage of RIP5-positive cells showed a significantly higher area percentage score in CTRL compared to DU (*p* = 0.0361) and DYS (*p* = 0.0082). The HYP had the lowest area percentage score (F (3, 36) = 4,481; Figure 5a).

The percentage of RIP5-positive cells in DU, HYP, and DYS kidneys gradually reduced over time. When formally tested for a linear trend among developmental periods, the HYP analysis did not show significance (R^2^ = 83.35%, β = −0.199 ± 0.162; Figure 5b). The overall expression of RIP5 in DU and DYS showed an exponential decline with the developmental age, with peak expression at 24 and 27 dw (R^2^ = 100%, R^2^ = 45.12%). On the other hand, in control, RIP5 demonstrated a linear growth with time (R^2^ = 99.97%, β = 0.028 ± 0.0007).

The RT−qPCR analysis demonstrated the *RIP5* mRNA expression in all tissues tested with no significant difference in the expression score comparing the CAKUT-affected kidneys and control (*p* = 0.7149, F (9, 4) = 1,535; Figure 3e). The *RIP5* mRNA expression score in different CAKUT phenotypes was not different compared to controls (F (3, 12) = 0.3483; Figure 3f).

## 3. Discussion

CAKUT contributes to 23% of birth abnormalities [1,2] and accounts for approximately half of pediatric cases and 7% of adult cases of the end-stage renal disease around the globe [3,4]. So far, monogenic disorders related to CAKUT involve mutations in 23 genes and have been identified in a small percentage (12%) of examined cases, with insufficient evidence of their causative role [7]. This research aimed to investigate the spatio-temporal expression of *RIP5*, a new CAKUT candidate [9], and *FGFR1*, and *FGFR2*, well-known potential candidate genes, in developing human kidneys and kidneys affected with congenital anomalies [12,13].

Human kidney morphogenesis, development, and maturation are complex processes finely regulated by the interaction of numerous genes. Among them, the expression of *FGFR1*, *FGFR2*, and *RIP5* [10,12,15,24,32,33] suggests that they all play an important role in nephrogenesis and nephron progenitor survival. Namely, in mice, deletion of *Fgfr1* or *Fgfr2* causes early embryonic lethality, while targeted disruption of *Fgfr1* and *Fgfr2* leads to CAKUT [10,25,26,27,28].

RIP5 knockdown in human embryonic renal cells inhibited FGF-stimulated phosphorylation of ERK, the main effector of FGF-induced transcriptional activity. This information suggests that RIP5 is involved in the downstream regulation of FGF signaling [9]. The results of our study demonstrate the co-expression of RIP5 and FGFR1 in the epithelial cells of convoluted tubules and RIP5 and FGFR2 in glomeruli, proximal, and distal convoluted tubules. Our earlier studies on human kidneys showed RIP5 expression in early human kidney development from the 5th to 22nd dw, noticed first in the undifferentiated metanephric mesenchyme [12]. We found a notable membrane-associated distribution of RIP5 in mesenchymal-derived cells in mice kidney development (E15.5) [9,13]. We also showed RIP5, FGFR1, and FGFR2 expression patterns, which decreased during human kidney development from the 6th to the 22nd developmental week [12]. However, although the percentage of positive cells was very low in this study, we found a more prominent expression pattern in parietal cells of immature glomeruli than in the renal vesicle stage. In the juxtaglomerular region, strong expression of RIP5 was noticed in the apical cytoplasm of convoluted tubules and endothelium of blood vessels but milder in glomeruli, with notable staining in visceral epithelial cells.

Expression dynamics and progression of the percentage of the RIP5-positive cells in DU, HYP, and DYS kidneys revealed a gradual decrease over observed dw, as opposed to CTRL, which demonstrated a linear growth with time. The significance of RIP5 in the initial stages of nephrogenesis and maturation and, along with FGFR1 and FGFR2, in vasculogenesis is already familiar [12,13]. We hypothesize that the expression of RIP5 during normal human kidney development reduces temporarily due to urine production and increases later since it undertakes additional functions in the maturation of the postnatal kidney and homeostasis.

The biological function of RIP5 is still mostly unknown. A study by Zha et al. found that overexpressing RIP5 causes cell death with characteristic DNA fragmentation, a hallmark of apoptotic cell death, via both caspase-dependent and caspase-independent apoptotic pathways [34]. In the study of Zhong et al., the downregulation of RIP5 (DSTYK) expression accelerated lung cancer cell growth and colony formation [35]. Our study demonstrated that expression dynamics of RIP5 in CAKUT-affected kidneys exhibit a divergent trend—a downregulation of RIP5 expression during the investigated developmental period—which could not be related to the expected levels of apoptosis in hypoplastic and dysplastic kidneys, indicating that other signaling pathways trigger the process of apoptosis. Further work will be needed to elucidate the molecular mechanisms responsible for the aforementioned decrease in RIP5 expression in the CAKUT kidneys.

Several genetic disorders responsible for structural kidney disease have been studied recently, including mutations in FGFRs [16,17,18,19]. Since FGFR activation results in an anti-apoptotic, pro-proliferative, and pro-survival response [15,36,37], it is reasonable to hypothesize about the altered cell response caused by CAKUT.

The CAKUT-affected kidneys demonstrated strong diffuse FGFR1 staining of the apical membrane of proximal convoluted tubules, similarly to Shima et al., who noticed strong immunoreactivity in the epithelium of the primitive tubules of dysplastic kidneys [38]. Interestingly, in the DYS, we noticed localization of FGFR1 in the nuclei of epithelial cells of convoluted tubules. According to Xie et al., nuclear localization of FGFRs can be found in diverse pathophysiological conditions and multiple tissues. Once within the nucleus, FGFRs can stimulate gene expression through many mechanisms, even epigenetically. Nuclear FGF/FGFR-mediated regulation of transcription proposes an alternative mechanism through which FGFs/FGFRs can directly elicit specific and rapid gene expression changes [39]. The analysis of the area percentage of FGFR1-positive cells has demonstrated a significant decrease in control compared to CAKUT phenotypes. Following area percentage analysis, the RT−qPCR analysis revealed a significantly higher FGFR1 expression score in the CAKUT-affected kidneys than in control. The expression of FGFR1 in HYP and DU kidneys increased during dw, unlike in control and DYS, which followed a quadratic trend with peak expression at the 27th dw. Namely, the 27th dw, the third developmental period of the kidney, is characterized by the generation of ampullae by local cell proliferation and the formation of new nephrons in the outer cortex. The nuclear localization of FGFR1, elevated the expression score of FGFR1 mRNA, increased the area percentage of FGFR1-positive cells in the cortex of DYS kidneys, and the overall decrease in the expression after the peak at the 27th developmental week in DYS suggest the altered expression pattern and protein function is in response to CAKUT pathophysiology.

FGFR2 immunoexpression was observed as strong, diffuse membranous and cytoplasmic staining patterns noticed in convoluted tubules, endothelial cells of blood vessels, and parietal epithelium of the CAKUT-affected kidneys. This staining pattern is correlated with the study of Shima et al., where strong immunoreactivity in the epithelium of the primitive tubules of the DYS was noticed [38]. Once more, we noticed localization of FGFR2 in the nuclei of epithelial cells of convoluted tubules in the DU and HYP kidneys. The area percentage of FGFR2-positive cells significantly differed between the cortex of CAKUT-affected kidneys and controls, where we noted the significantly lower area percentage score. The percentage of FGFR2-positive cells in DU, HYP, and DYS gradually reduced over time, whereas controls demonstrated a positive, stable plateau of expression from the 22nd to the 38th dw. These results highlight the essential role of FGFR2 in nephrogenesis and demonstrate its importance in postnatal kidney function maintenance during normal human development.

In the study of Tsimafeyeu et al., overexpression of FGFR2 has been associated with poor survival and resistance to first-line systemic therapy in papillary renal cell carcinoma [40]. ERK1/2 is a major downstream mediator of FGF/FGFR signaling. The pathophysiology of acute and chronic kidney injury is mediated by the ERK1/2 signaling pathway, which also regulates the response of renal cells to a wide range of stimuli [41]. Another study on mice underlines the important role of ERK in forming cysts resulting from the tubular epithelium’s aberrant proliferation in AD polycystic kidney disease [42]. The RT−qPCR analysis revealed a significantly higher *FGFR2* expression score in the CAKUT-affected kidneys than in the control group. When we compared the *FGFR2* mRNA expression in different CAKUT phenotypes to controls, we detected a significantly higher expression score in HYP than in control. A statistically significant *FGFR2* mRNA expression score in the HYP kidneys relates to the higher importance of the ERK signaling pathway in the repair mechanism. Although the *FGFR2* mRNA expression score in the DU is not significant, it is higher than CTRL, and this increase could be a consequence of the repair mechanism mediated by ERK1/2 attempting to repair the defect.

As stated above, the majority of data constituting the basis of our current knowledge on the expression and related function of candidate genes in CAKUT kidney tissues are derived from gene targeting studies in knockout mice. To the best of our knowledge, our investigation is the first in the existing literature to evaluate the protein expression of these candidate target genes in the observed developmental periods. It is also one of a few studies overall concerning the expression of other CAKUT candidate genes in the same spectrum of renal diseases. The study of Tokat et al., demonstrated significantly decreased expression levels of CAKUT candidate genes, Glial cell line-derived neurotrophic factor/ Ret proto-oncogene (GDNF/RET), FGFR2, and Paired box gene 2 (PAX2) proteins in tissues taken from patients with primary VUR [43]. Another study states that the overexpression of PAX2 is linked to cancer, while underexpression causes malformed ureters and hypoplastic kidneys [44]. The study of Jain et al., conducted on tissues with congenital renal dysplasia using a microarray analysis, has shown decreased expression of genes known to be essential for normal kidney development, such as bone morphogenetic protein 7 (BMP7), angiotensin receptor 2 (AGTR2), and SAL-like 1 (SALL1) [45].

Advancements in research on animal models have led to the identification of numerous genes necessary for control of renal differentiation. However, some of these genes are not expressed in mice or have different paralogs. Considering this, analysis of human samples is vital to understanding the normal expression pattern of CAKUT candidate genes. The occurrence of these proteins in kidneys and their expression dynamics suggest that FGFR1, FGFR2, and RIP5 play critical roles in nephrogenesis, nephron progenitor survival, the regulation of overall homeostasis, and the maturation of kidney structures during postnatal development. The influence of CAKUT candidate genes on the nephrogenesis and function of the kidney is supported by statistically significant variations in the spatio-temporal expression patterns of the examined markers. The discovery that the expression of *FGFR1* and *FGFR2* mRNA and their proteins were significantly higher in CAKUT-affected kidneys than in normal tissue in both the cytoplasmic and nuclear regions, as well as deranged expression dynamics of the examined proteins, suggests that the activation of the FGF pathway is a critical event in the pathogenesis of the congenital anomalies of kidney spectrum. A downregulation of RIP5 expression during the investigated developmental period could not be attributed to the expected levels of apoptosis in HYP and DYS, indicating that the process of apoptosis may be triggered via other signaling pathways. Our findings highlight their importance in normal and pathological kidney development.

Further work will be needed to assess and reveal their role in regulating kidney differentiation, which might improve the precision medicine approach to congenital kidney and urinary tract anomalies.

## 4. Materials and Methods

### 4.1. Tissue Procurement and Processing

A total of 19 paraffin blocks of fetal kidney tissue (Table 1) from spontaneous miscarriages and eugenic abortions due to severe abnormalities, were sampled from the Department of Pathology at the University Hospital Center Split and processed with the permission of the Ethical and Drug Committee of the University Hospital in Split (class: 003-08/16-03/0001, approval number: 2181-198-03-04-16-0024) following the Helsinki Declaration [46]. The specimens without maceration were included in the study. External measurement (crown–rump length) and menstrual data were used to estimate the gestational age [47]. Kidney pathology was classified by gross morphology and routine histopathology.

After the post-mortem section, renal tissue was fixed in buffered formalin (4% paraformaldehyde in 0.1 M phosphate buffer saline, PBS). After dehydration in graded ethanol solutions and clearing in xylol, the tissue was embedded in paraffin blocks, serially cut to a thickness of 5 µm on a microtome, and mounted on slides. Every tenth section was stained by hematoxylin–eosin (H&E). Proper tissue preservation, stages in normal fetal kidney development, and pathological findings in the kidneys with CAKUT were examined by light microscopy.

### 4.2. Immunofluorescence

Following deparaffinization in xylol and rehydration in graded water–ethanol solutions, histological slides were cooked in a water steamer in 0.01 M citrate buffer (pH 6.0) for 30 min at 95 °C and subsequently cooled to room temperature. After washing in 0.1 M PBS, a protein-blocking solution (ab64226, Abcam, Cambridge, UK) was applied for 20 min to inhibit nonspecific staining. Primary antibodies were applied and incubated overnight in a humidity chamber, rinsed with PBS, and incubated with secondary antibodies for one hour (Table 2). After rinsing in PBS, DAPI (4′,6-diamidino-2-phenylindole) was applied to visualize nuclei. Slides were rinsed in PBS, covered by mounting media (Immuno-Mount, Thermo Shandon, Pittsburgh, PA, USA), and a coverslip.

The preadsorption test was conducted so that each primary antibody was diluted in a blocking solution at the predetermined concentration. Sections were treated with a solution to which an appropriate peptide antigen was added. No evidence of antibody staining was detected. No nonspecific binding of secondary antibodies or false-positive results was detected when primary antibodies were omitted from the immunofluorescence protocol.

### 4.3. Data Acquisition

H&E slides were analyzed by a light microscope (BX40, Olympus, Tokyo, Japan). Images of the fetal kidney cortex (nephrogenic zone and juxtamedullary region) were captured by an epifluorescence microscope (BX51, Olympus, Tokyo, Japan) equipped with a Nikon DS-Ri2 camera (Nikon Corporation, Tokyo, Japan) with NIS-Elements F software. FGFR1, FGFR2, and RIP5 were analyzed in ten non-overlapping representative fields at ×40 magnification with constant exposure time. Diffuse or punctate green staining was interpreted as positive FGFR1 and FGFR2, and red staining as positive RIP5. 

### 4.4. Image Analysis of Area Percentage

The photomicrographs were processed and evaluated with ImageJ software (National Institutes of Health, Bethesda, MD, USA) for quantitative cell evaluation of immunoreactivity as described previously [48,49]. Firstly, the fluorescence leakage was reduced by subtracting the red countersignal from the green fluorescence, and then a median filter with a 2.0-pixel radius was used. Each image was subsequently adjusted by the threshold method (triangle thresholding algorithm method), and the fluorescence percentage area was measured using the “analyze particles” option. Considering inter-observer variations, three expert histologists analyzed the captured microphotographs independently, setting the background thresholds using negative control images. Interrater agreement was proven with interclass correlation analysis, which yielded a coefficient > 0.8, indicating excellent agreement [50]. 

### 4.5. Statistical Analysis of Area Percentage

GraphPad Prism 9.0.0 software was used for statistical analyses (GraphPad Software, San Diego, CA, USA). The Shapiro–Wilk test was used to check normal distribution.

Each dataset regarding area percentage analysis was described with p at the probability level of *p* < 0.05 being regarded as statistically significant and the F distribution, F (DFn, Dfd) where DFn marks the degrees of freedom numerator and Dfd degrees of freedom denominator. The percentage of positive cells was expressed as the mean ± standard deviation (SD).

For studying expression dynamics and trends of different proteins through developmental periods, linear and nonlinear regression modeling was used. A coefficient in models used for trend description is presented as point estimate ± standard error. The coefficient of determination (R^2^) was used as a goodness of fit measure. A linear trend was described using the slope of a linear regression line (β).

All graphs were created using GraphPad Prism 9.0.0. Plates were assembled using Adobe Photoshop (Adobe, San Jose, CA, USA). Microphotographs were processed for background subtraction and contrast for presentation purposes.

### 4.6. RNA Isolation and qRT-PCR

To analyze CAKUT candidate genes, we used RNA isolated from CTRL (*n* = 4), DU (*n* = 3), HYP (*n* = 3), and DYS (*n* = 5) phenotypes. RNA isolation was performed according to the Invitrogen protocol with the PureLink™ FFPE RNA Isolation Kit (Cat. No. K156002, Invitrogen, Waltham, MA, USA). DNA digestion was performed using DNase I, Amplification Grade, according to the manufacturer’s instructions (Cat. No. 18068015 Invitrogen, Waltham, MA, USA). Examination using a UV spectrophotometer gave a value for optical density (OD)260/OD280 of 1.69–2.01). Reverse transcription was carried out with 2 µg of total mRNA using a High-Capacity cDNA Reverse Transcription Kit according to the manufacturer’s instructions (Cat. No. 4368814, Applied Biosystems, Waltham, MA, USA) and analyzed by qPCR using Taq™ Universal SYBR^®^ Green Supermix (Cat. No. 1725121, BioRad, Hercules, CA, USA) on a BioRad CFX96 Real-Time System (C1000 Touch Thermal Cycle). Primer sequences are listed in Table 3. *GAPDH* and *PPIA* were used as the housekeeping genes. The non-template control (NTC) sample comprised every component of the master mix, excluding the cDNA sample. Gene expression was normalized to NTC and analyzed using the comparative Ct method. The Shapiro–Wilk test was used to check for a normal distribution. An unpaired *t*-test and unpaired *t*-test with Welch’s correction were used to determine the significance level between the CAKUT kidneys and CTRL group. Ordinary one-way ANOVA with Tukey’s multiple comparisons test was used to determine the significant differences in the mRNA expression of observed genes between different CAKUT phenotypes and CTRL. Each dataset regarding RT−qPCR analysis was described, with *p* at the probability level of *p* < 0.05 being regarded as statistically significant and the F distribution, F (DFn, Dfd) where DFn marks the degrees of freedom numerator and Dfd degrees of freedom denominator.

## Figures and Tables

**Table 1:** The samples of human fetal kidneys (*n* = 19) analyzed in the study.

Gestational Age/Developmental Weeks	Number of Kidney Samples	Renal and Associated Pathology
22	2	Normal kidneys (CTRL)
27	2	
35	1	
38	1	
22	2	Dysplastic kidneys (DYS)
27	1	
35	1	
38	3	
30	1	Hypoplastic kidneys (HYP)
37	1	
38	1	
24	1	Duplex kidneys (DU)
30	1	
41	1	

**Table 2:** Primary and secondary antibodies used for immunofluorescence.

Antibodies		Catalog Number	Host	Dilution	Source
Primary	Flg (C-15)/FGFR1	sc-121	Rabbit	1:50	Santa Cruz Biotechnology (Texas, TX, USA)
	Bek (C-17)/FGFR2	sc-122	Rabbit	1:50	Santa Cruz Biotechnology (Texas, TX, USA)
	RIP5 (N-16)	sc-162109	Goat	1:50	Santa Cruz Biotechnology (Texas, TX, USA)
Secondary	Rhodamine Red™-X (RRX) AffiniPure Anti-Goat IgG (H + L)	705-295-003	Donkey	1:300	Jackson Immuno Research Laboratories, Inc., (Baltimore, PA, USA)
	Alexa Fluor^®^488 AffiniPure Anti- Rabbit lgG (H + L)	711-545-152	Donkey	1:300	Jackson Immuno Research Laboratories, Inc., (Baltimore, PA, USA)

**Table 3:** Primers used in RT−qPCR.

Gene Locus	Forward Primer (5′-3′)	Reverse Primer (5′-3′)
*FGFR 1*	CGCCCCTGTACCTGGAGATCATCA	TTGGTACCACTCTTCATCTT
*FGFR 2*	GCCTGGAAGAGAAAAGGAGATTAC	GGATGACTGTTACCACCATACA
*RIP5*	TTGCATACTGATCCTCGG	TGTGCACTAGTTCATACT
*GAPDH*	GAAGGTGAAGGTCGGAGTC	GAAGATGGTGATGGGATTTC
*PPIA*	ACCGCCGAGGAAAACCGTGTA	TGCTGTCTTTGGGACCTTGTCTGC

## Data Availability

All data and materials are available upon request.
